# Obesity Predicts Longer Operative Time and Worse Functional Recovery After Total Knee Arthroplasty Under Enhanced Recovery Protocols in Asian Patients

**DOI:** 10.7759/cureus.88391

**Published:** 2025-07-20

**Authors:** Ramesh Radhakrishnan, Akshay Padki, Sheng Xu, Audrey Xinyun Han, Xuan Liu, Ming Han Lincoln Liow, Darren Tay Keng Jin, Seng Jin Yeo, Jerry Chen, Hee Nee Pang

**Affiliations:** 1 Orthopaedic Surgery, Singapore General Hospital, Singapore, SGP

**Keywords:** asian population, bmi, eras, functional outcomes, knee society knee score, obesity, oxford knee score, range of motion, total knee arthroplasty

## Abstract

Background

Obesity is a growing global health concern that poses challenges to optimizing outcomes following total knee arthroplasty (TKA). While Enhanced Recovery After Surgery (ERAS) protocols aim to improve perioperative recovery, it remains unclear whether they sufficiently mitigate the adverse impact of obesity, particularly in Asian populations with differing body composition and recovery patterns.

Methods

A retrospective cohort study was conducted at a tertiary academic center involving patients who underwent primary TKA under a standardized ERAS protocol between January 2020 and December 2021. Patients were stratified into three body mass index (BMI) categories: non-obese (<30 kg/m²), obese class I (30-34.9 kg/m²), and obese class II+ (≥35 kg/m²). Functional outcomes at six months, including Knee Society Knee Score (KSKS), Knee Society Functional Score (KSFS), Oxford Knee Score (OKS), range of motion (ROM), and patient-reported measures, were analyzed across BMI groups. Multivariable linear regression was used to adjust for age, gender, American Society of Anesthesiologists (ASA) classification, and Charlson Comorbidity Index (CCI).

Results

A total of 334 patients were included (non-obese: n = 245; obese class I: n = 65; obese class II+: n = 24). Patients with BMI ≥35 had significantly lower six-month OKS (β = -3.89, p = 0.013), KSFS (β = -6.68, p = 0.001), and reduced ROM in extension (β = -9.95°, p = 0.005) compared to the non-obese group. Obese class I patients had significantly lower KSFS (β = -2.30, p = 0.004), though OKS and ROM extension were not statistically different. No significant differences were found in surgical complication rates, infection, readmission, or revision. Surgical duration trended longer with increasing BMI but did not reach statistical significance.

Conclusion

Obesity, particularly BMI ≥35, is an independent predictor of inferior functional outcomes and reduced knee extension following TKA, even within a standardized ERAS pathway. These findings underscore the need for targeted preoperative optimization and rehabilitation strategies for obese patients undergoing TKA.

## Introduction

Total knee arthroplasty (TKA) remains a definitive surgical intervention for end-stage knee osteoarthritis, offering substantial pain relief and functional restoration [1]. However, the growing prevalence of obesity poses a critical challenge to optimizing outcomes following TKA. Obesity is widely recognized as a modifiable risk factor associated with increased perioperative morbidity, prolonged operative time, and delayed postoperative recovery [2]. While previous studies have explored the impact of body mass index (BMI) on TKA outcomes, the independent effect of obesity on functional recovery within Enhanced Recovery After Surgery (ERAS) frameworks, particularly in the Asian population, remains inadequately characterized [3,4].

ERAS protocols have transformed perioperative care by standardizing multimodal strategies to minimize surgical stress, promote early mobilization, and shorten hospital stay [5,6]. Despite these advances, it is unclear whether ERAS pathways sufficiently mitigate the adverse impact of obesity on functional outcomes after TKA [7]. Furthermore, the majority of existing literature is derived from Western populations, with limited data on how BMI influences recovery and surgical metrics in Asian cohorts, who often differ in body composition, comorbidity profiles, and cultural attitudes toward rehabilitation [8]. This gap in knowledge underscores the necessity for targeted research that investigates the specific effects of obesity on TKA outcomes within Asian populations, particularly in the context of ERAS protocols. Understanding these dynamics could lead to tailored interventions that enhance recovery and optimize surgical results for obese patients undergoing TKA [9].

This study aims to evaluate the influence of obesity on perioperative and functional outcomes following primary TKA performed under standardized ERAS protocols in an Asian tertiary care setting. We hypothesize that obesity is an independent predictor of prolonged operative duration and inferior functional outcomes at six months postoperatively, despite adherence to ERAS principles. Clarifying this relationship may inform preoperative risk stratification and guide tailored perioperative optimization strategies in this growing patient demographic.

## Materials and methods

Study design and setting

This was a retrospective cohort study conducted at a tertiary academic medical center. The study included patients who underwent primary TKA between January 2020 and December 2021, all of whom were managed under an established ERAS protocol. The study received ethical approval from the SingHealth Centralised Institutional Review Board (CIRB Ref No.: 2023/2725) and adhered to the principles outlined in the Declaration of Helsinki. Due to its retrospective nature, the requirement for informed consent was waived.

Patient selection

Patients were eligible if they were diagnosed with symptomatic knee osteoarthritis, underwent primary TKA, and completed the institutional ERAS protocol. Inclusion criteria included complete preoperative and postoperative (six months) documentation of all parameters, especially BMI and clinical outcomes. Exclusion criteria were prior revision TKA, perioperative complications requiring deviation from ERAS, or incomplete clinical records. Patients were categorized into three groups with BMI criteria based on the WHO guidelines, namely, non-obese (<30), obese class I (30-34.9), and obese class II (35-39.9).

Perioperative protocol and surgical technique

All surgeries were performed by fellowship-trained orthopedic surgeons utilizing a standardized medial parapatellar approach. Prosthesis choice, use of cemented versus cementless implants, and cementing technique were at the discretion of the attending surgeon but followed institutional guidelines. Perioperative management adhered strictly to the ERAS protocol, incorporating preoperative patient education, multimodal analgesia, early ambulation (within 24 hours post surgery), and physiotherapy-guided rehabilitation starting on the day of surgery. This is followed up with regular home visits by the therapists to continue physiotherapy sessions from the comfort of patients' homes.

Data collection

Data were collected from electronic medical records and institutional databases. The variables included demographic information (age, sex, ethnicity, BMI, comorbidities, Charlson Comorbidity Index (CCI), and American Society of Anesthesiologists (ASA) classification), surgical details (duration of surgery and length of hospital stay), preoperative range of motion (ROM) (ROM-flexion (ROME) and ROM-extension (ROMS) measured by physiotherapists using handheld goniometers), and postoperative outcomes. Postoperative data included BMI, ROM, Knee Society Knee Score (KSKS), Knee Society Function Score (KSFS), Oxford Knee Score (OKS), Physical Component Summary (PCS), Mental Component Summary (MCS), patient satisfaction (SAT), and expectation fulfillment (EXP) assessed at six months postoperatively.

Outcome measures

The primary outcomes were KSKS and KSFS. Secondary outcomes included postoperative ROM (flexion and extension), other patient-reported outcomes (OKS, PCS, and MCS), as well as changes (Δ) in functional scores from baseline to six months and patient-reported satisfaction and expectations.

## Results

In this cohort, statistically significant differences were observed in patient age and comorbidity burden across BMI categories. Patients in the higher BMI groups tended to be younger (p = 0.001) and exhibited lower CCI scores (p = 0.022), suggesting a trend toward earlier surgical intervention in individuals with obesity. Gender distribution and ASA classification were comparable across groups, with no significant differences noted (p > 0.05). These findings indicate that while obesity is associated with younger surgical age and fewer comorbidities, baseline surgical risk remains consistent regardless of BMI classification (Table 1).

**Table 1:** Patient demographics. ASA: American Society of Anesthesiologists classification; CCI: Charlson Comorbidity Index.

Demographic variable	Non-obese (<30)	Obese class I (30–34.9)	Obese class II+ (≥35)	P-value
Number of patients (n)	245.0	65.0	24.0	-
Mean age	68.1	65.6	63.5	0.001
Female count	152.0	46.0	18.0	0.281
Male count	95.0	21.0	6.0	0.281
Mean CCI	2.66	2.49	2.04	0.022
Mean ASA	2.08	2.03	2.25	0.003

The comparative analysis of postoperative outcomes across BMI categories demonstrated several notable trends. Patients in the obese class II+ group experienced significantly inferior functional recovery, as reflected by lower six-month KSFS (67.5 vs. 77.4; p = <0.001) and OKS scores (38.5 vs. 41.1; p = 0.008), despite undergoing surgery under ERAS protocols. Additionally, a significant difference was observed in postoperative range of motion in extension, with the obese class II+ group demonstrating poorer extension at six months (p = 0.004). Although the mean surgical duration increased with BMI (from 87.5 to 96.7 minutes), this did not reach statistical significance (p = 0.08). Postoperative radiographic alignment and deformity correction were comparable across groups, with no significant differences observed. Similarly, six-month BMI, length of stay, time to ambulation, postoperative expectations, and satisfaction scores showed no meaningful variation. Rates of infection, 30-day readmission, and revision surgery were low and did not differ significantly among groups (Table 2).

**Table 2:** Comparison of postoperative functional, surgical, and patient-reported outcomes across BMI categories. KSFS: Knee Society Function Score; KSKS: Knee Society Knee Score; OKS: Oxford Knee Score; MCS: Short Form-36 Mental Component Summary; PCS: Short Form-36 Physical Component Summary; ROM: range of motion; BMI: body mass index.

Outcome measure	Non-obese (<30)	Obese class I (30–34.9)	Obese class II+ (≥35)	P-value
Surgical duration	87.5	90.2	96.7	0.08
Length of stay	1.14	1.05	1.25	0.676
6-month KSFS	77.4	67.9	67.5	<0.001
6-month KSKS	86.6	82.8	81.3	0.071
6-Month OKS	41.1	38.9	38.5	0.008
6-month MCS	59.1	57.5	55.1	0.087
6-month PCS	48.4	46.3	46.6	0.236
6-month ROM extension	111.8	111.1	100.5	0.004
6-month ROM flexion	3.38	4.09	3.5	0.576
6-month degree of coronal deformity	4.53	4.36	5.15	0.37
6-month BMI	24.5	31.1	37.2	< 0.001
Postoperative expectation	2.21	2.26	2.3	0.934
Postoperative satisfaction	2.28	2.19	2.2	0.812
Ambulation time (hours)	11.4	12.6	13.8	0.682
Infection rate	0.016	0.015	0	0.821
30-day readmission	0.024	0.03	0.042	0.866
Revision surgery	0.012	0.015	0	0.842

Multivariable regression analyses

Multivariable linear regression analyses identified BMI as an independent predictor of postoperative functional outcomes following TKA under ERAS protocols. After adjusting for age, gender, ASA classification, and comorbidity burden (CCI), patients in the obese class II+ group (BMI ≥35) had significantly lower OKS (β = -3.89, p = 0.013) and KSFS (β = -6.68, p = 0.001) at six months compared to the non-obese reference group. Obese class I patients (BMI = 30-34.9) also demonstrated a significant reduction in KSFS (β = -2.30, p = 0.004), though the decline in OKS (β = -2.13, p = 0.063) did not reach statistical significance.

In terms of postoperative mobility, patients in the obese class II+ group experienced a nearly 10° reduction in extension range of motion at six months compared to the non-obese group (β = -9.95, p = 0.005). This was the only BMI category associated with significantly impaired ROM extension. Age, ASA, and CCI were not significant predictors in any of the three models, and gender showed no consistent effect (Tables 3-5).

**Table 3:** Multivariable linear regression analysis of six-month Oxford Knee Score (OKS). ASA: American Society of Anesthesiologists classification; CCI: Charlson Comorbidity Index.

	Coef.	Std. error	t	P > |t|	95% CI	
					0.025	0.975
Intercept	40.4	4.75	8.51	0.0	31.1	49.8
Obese class I (30–34.9)	-2.3	0.797	-2.89	0.004	-3.87	-0.735
Obese class II+ (≥35)	-2.24	1.27	-1.76	0.079	-4.75	0.265
Gender (male)	1.76	0.654	2.7	0.007	0.478	3.05
Age	0.056	0.065	0.864	0.388	-0.072	0.184
CCI	-0.389	0.426	-0.912	0.362	-1.23	0.45
ASA	-1.28	1.21	-1.06	0.288	-3.66	1.09

**Table 4:** Multivariable linear regression analysis of six-month Knee Society Functional Score (KSFS). ASA: American Society of Anesthesiologists classification; CCI: Charlson Comorbidity Index.

	Coef.	Std. error	t	P > |t|	95% CI	
					0.025	0.975
Intercept	92.7	15.1	6.13	0.0	62.9	123.0
Obese class I (30–34.9)	-10.3	2.54	-4.07	0.0	-15.3	-5.33
Obese class II+ (≥35)	-9.24	4.06	-2.28	0.023	-17.2	-1.26
Gender (male)	6.73	2.08	3.23	0.001	2.63	10.8
Age	-0.083	0.207	-0.4	0.69	-0.49	0.324
CCI	-0.572	1.36	-0.422	0.673	-3.24	2.1
ASA	-5.04	3.84	-1.31	0.191	-12.6	2.52

**Table 5:** Multivariable linear regression analysis of six-month range of motion in extension. ASA: American Society of Anesthesiologists classification; CCI: Charlson Comorbidity Index.

	Coef.	Std. error	t	P > |t|	95% CI	
					0.025	0.975
Intercept	103.0	13.5	7.63	0.0	76.3	129.0
Obese class I (30–34.9)	-0.667	2.24	-0.298	0.766	-5.08	3.74
Obese class II+ (≥35)	-9.95	3.49	-2.85	0.005	-16.8	-3.09
Gender (male)	2.76	1.81	1.52	0.129	-0.807	6.32
Age	0.13	0.184	0.705	0.481	-0.233	0.493
CCI	0.436	1.19	0.367	0.714	-1.9	2.77
ASA	-0.934	3.38	-0.277	0.782	-7.59	5.72

These findings confirm that obesity, particularly at a BMI ≥35, is an independent determinant of poorer functional and mobility outcomes following TKA, even when managed under a standardized ERAS pathway.

## Discussion

This study evaluated the impact of obesity on postoperative outcomes following TKA under a standardized ERAS protocol in an Asian population. Our findings demonstrate that obesity, particularly at higher BMI thresholds, is independently associated with inferior functional outcomes and reduced ROM, despite adherence to modern perioperative optimization strategies.

Patients in the obese class II+ group (BMI ≥35 kg/m²) experienced significantly poorer outcomes at six months, including lower OKS and KSFS scores compared to their non-obese counterparts. This is consistent with existing literature [10-14]. McElroy et al. also observed lower postoperative mean Knee Society objective and function scores for morbidly obese patients (40 ≤ BMI < 50 kg/m^2^) [15]. This association persisted after adjusting for age, gender, comorbidities, and ASA classification, highlighting obesity as an independent risk factor for suboptimal recovery. This remained true in a long-term study by Liljensøe et al., who found that high BMI increases the risk of poor quality of life (Short Form-36) and physical function (KSS) up to three to five years postoperatively [16]. Additionally, patients in this group demonstrated significantly reduced knee extension postoperatively, with a nearly 10° loss in ROM extension relative to the non-obese group. This finding is particularly clinically relevant, as extension deficits are known to impair gait mechanics and delay functional independence. Similarly, Palanne et al. found that a one-unit increase in body fat percentage decreased postoperative ROM by 0.37° [17].

Interestingly, while obese class I (BMI: 30-34.9 kg/m²) patients also exhibited reduced KSFS, their OKS and ROM extension were not significantly different from those of the non-obese group. This suggests a graded relationship between BMI and outcome severity, with more pronounced deficits emerging at higher levels of obesity. Notably, surgical duration trended longer in higher BMI groups, although this did not reach statistical significance, possibly due to intraoperative standardization under ERAS pathways mitigating procedural delays.

There was no statistically significant difference in rates of infection, 30-day readmission, and revision surgery among groups, which is consistent with multiple existing studies that found increasing BMI was not associated with increased rate of complications in the short term [18-22]. However, there are multiple existing studies with longer-term follow-ups, showing a positive correlation between increasing BMI and the rate of complications [23-25]. Further studies with a larger sample size and a longer duration of follow-up are warranted to accurately assess the relationship between BMI and associated postoperative complications.

A key strength of this study is its focus on an Asian cohort, a population often underrepresented in orthopedic outcome research. Asian patients differ in body composition and cultural approaches to recovery, making these findings particularly valuable for regional surgical planning and counseling. Furthermore, this study benefits from a relatively large sample size and the inclusion of functional, radiographic, and patient-reported outcomes, offering a comprehensive assessment of recovery quality.

However, several limitations warrant consideration. First, the sample size of the obese class II+ group was limited (n = 24), which may affect the generalizability of findings within this subgroup. Second, although multivariable regression was used to adjust for confounders, residual confounding due to unmeasured variables, such as use of cemented versus cementless implants, use of cruciate retaining vs. posterior stabilized inserts, nutritional status, muscle mass, or adherence to rehabilitation, may still be present. Third, the study design is retrospective in nature, which inherently limits causal inference. Lastly, long-term outcomes, including implant survivorship beyond six months, were not evaluated. Future longitudinal studies are needed to determine whether these early deficits persist or widen over time.

## Conclusions

In summary, this study reinforces the detrimental effect of obesity, particularly BMI ≥35, on functional and biomechanical outcomes following TKA, even within a standardized ERAS framework. These findings suggest a need for tailored perioperative strategies and preoperative optimization in obese patients, including weight management and targeted physiotherapy, to enhance recovery trajectories and surgical success.
